# Transcriptional ITPR3 as potential targets and biomarkers for human pancreatic cancer

**DOI:** 10.18632/aging.204080

**Published:** 2022-05-17

**Authors:** Wangyang Zheng, Xue Bai, Yongxu Zhou, Liang Yu, Daolin Ji, Yuling Zheng, Nanfeng Meng, Hang Wang, Ziyue Huang, Wangming Chen, Judy Wai Ping Yam, Yi Xu, Yunfu Cui

**Affiliations:** 1 Department of Hepatopancreatobiliary Surgery, Second Affiliated Hospital of Harbin Medical University, Harbin 150086, China; 2 The Key Laboratory of Myocardial Ischemia, Harbin Medical University, Ministry of Education, Harbin 150086, China; 3 Department of Clinic of Internal Medicine I, Ulm University, Ulm 89081, Germany; 4 Department of Pediatric, Second Affiliated Hospital of Harbin Medical University, Harbin 150086, China; 5 Department of Hepatopancreatobiliary Surgery, Fourth Affiliated Hospital of Harbin Medical University, Harbin 150086, China; 6 Department of Pathology, Li Ka Shing Faculty of Medicine, The University of Hong Kong, Hong Kong 999077, China; 7 Department II of Gastroenterology, Third Affiliated Hospital of Harbin Medical University, Harbin 150086, China

**Keywords:** pancreatic cancer, bioinformatics analysis, ITPRs, therapeutic targets, biomarker

## Abstract

Inositol 1,4,5-Triphosphate Receptor Family (ITPRs) are necessary intracellular Ca2+-release channel encoders and participate in mammalian cell physiological and pathological processes. Previous studies have suggested that ITPRs participate in tumorigenesis of multiple cancers. Nevertheless, the diverse expression profiles and prognostic significance of three ITPRs in pancreatic cancer have yet to be uncovered. In this work, we examined the expression levels and survival dates of ITPRs in patients with pancreatic cancer. As a result, we identified that ITPR1 and ITPR3 expression levels are significantly elevated in cancerous specimens. Survival data revealed that over-expression of ITPR2 and ITPR3 resulted in unfavourable overall survival and pathological stage. The multivariate Cox logistic regression analysis showed that ITPR3 could be an independent risk factor for PAAD patient survival. Moreover, to investigate how ITPRs work, co-expressed genes, alterations, protein-protein interaction, immune infiltration, methylation, and functional enrichment of ITPRs were also analyzed. Then, we evaluated these findings in clinical samples. Moreover, the gain and loss of function of ITPR3 were also conducted. The electron microscope assay was employed to explore the role of ITPR3 in pancreatic cancer cell lines’ endoplasmic reticulum stress. In summary, our findings demonstrated that ITPR3 has the potential to be drug targets and biomarkers for human pancreatic cancer.

## INTRODUCTION

With a 5-year survival rate of about 6% [1], pancreatic cancer is one of the fatal tumors in the digestive system and has become the fourth leading cause of cancer-associated deaths worldwide [2]. Not until it reaches an advanced stage do most pancreatic cancer patients have any typical symptoms [3]. Although surgical management is the hope of a cure, most patients are not surgical candidates due to their high tumor stage. Even for the patients who underwent radical surgery, the one-year recurrence rate is still 54% [4]. Chemotherapy can hardly do anything for prolonging patients’ lifespan because of the chemoresistance. Therefore, elucidation of the molecular pathogenesis of pancreatic cancer may be crucial to conquer this lethal disease [5].

As a versatile second messenger, Ca2+ takes part in cell pathology and physiology processes, including cell inflammation, proliferation, differentiation, apoptosis, and autophagy. It has been proved to be associated with cancer development and progression [6, 7]. ITPRs family have three members: ITPR1, ITPR2 and ITPR3. Their receptor Inositol 1,4,5-trisphosphate receptors 1-3 (IP3R1-3) is one of the essential intracellular Ca2+-release channels [8]. Previous studies have shown that each type of IP3Rs has its tissue specificity: IP3R1 is highly expressed in the nervous system, IP3R2 in cardiomyocytes and hepatocytes, and IP3R3 in epithelial cells [9]. Thus, dysregulated ITPR1 is frequently found in neurological disorders such as Gillespie syndrome [10], Spinocerebellar ataxia [11], etc. ITPR2 mutations have been identified in exocrine deficiency [12] and amyotrophic lateral sclerosis [13]. For ITPR3, it is associated with Kawasaki disease development [14]. What’s more, these three members have already been proven to participate in some human cancers: ITPR1 in Sézary Syndrome and osteosarcoma [15, 16], ITPR2 in acute myeloid leukaemia and clear cell renal cell carcinoma [17, 18], ITPR3 in multiple cancers: cervical squamous cell carcinoma [19], glioblastoma [20], cholangiocarcinoma [21] and so on. Based on bioinformatics analysis, many potential cancer targets and new biomarkers have been identified and verified [22, 23]. As far as we know, bioinformatics analysis has yet been utilized to unveil the functions of the ITPRs in pancreatic cancer.

Therefore, we evaluated the expression profiles and prognostic value of ITPR1-3 in pancreatic cancer through data mining. Moreover, we try to explore several potential targets of ITPRs in pancreatic cancer by co-expressed genes and PPI analysis. And we analyzed immune infiltration, methylation, and functional enrichments of ITPRs for studying their potential functions in pancreatic cancer. The multivariate Cox logistic regression analysis showed that ITPR3 could be an independent risk factor for PAAD patient survival. Moreover, the gain and loss of function of ITPRs in pancreatic cancer cell lines was also conducted. As important intracellular Ca2+-release channels, the dysregulation of ITPR3 is accompanied by the disorder of cell homeostasis and causes endoplasmic reticulum stress, which has also been shown in GO analysis. We employed an electron microscope assay to explore the function of ITPRs in pancreatic cancer cell lines endoplasmic reticulum stress. Our study investigated both the molecular and potential mechanisms of ITPRs in pancreatic cancer, which may be helpful for further research on ITPRs.

## RESULTS

### ITPRs’ transcriptional levels are elevated in pancreatic cancer tissues and cell lines

**Figure 1:**
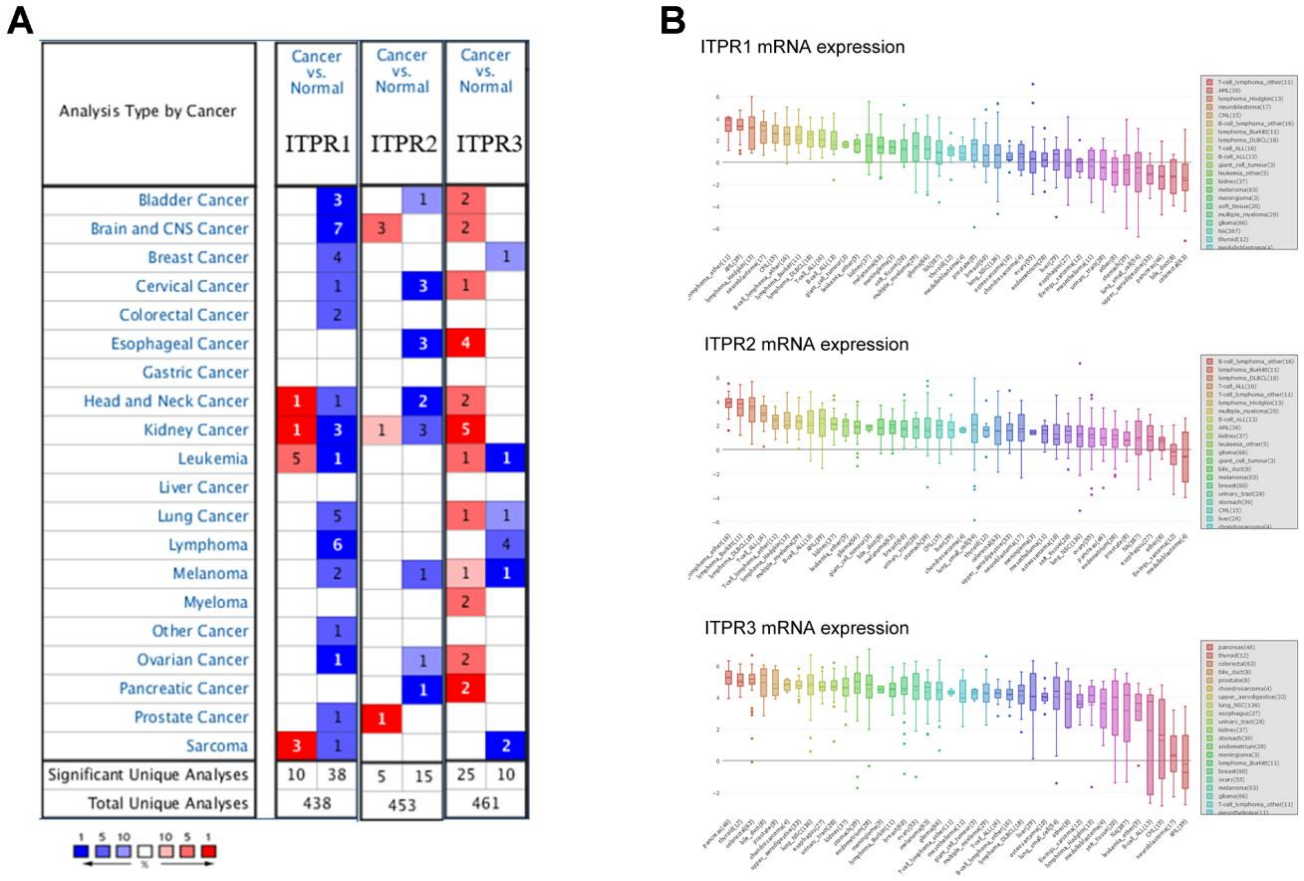
**The overview of expression of ITPRs in pan-cancer.** (**A**) The transcription levels of ITPRs in different types of cancers (ONCOMINE). (**B**) The expression of ITPRs in cancer cell lines (CCLE). Result of the expression of ITPR2 in cancer cell lines (CCLE). The expression of ITPR3 in cancer cell lines (CCLE).

Emerging evidence has indicated that ITPR1, ITPR2, and ITPR3 were universally dysregulated in many cancers. To identify the essential function of ITPRs, we compared ITPRs’ transcriptional levels by using ONCOMINE database. Figure 1A showed the significant datasets for ITPRs mRNA up-regulated (red) or down-regulated (blue) statistically. It was found that ITPR1 was suppressed in bladder cancer, breast cancer, colorectal cancer, and liver cancer while up-regulated in sarcoma and leukaemia. ITPR2 was up-regulated in brain and prostate cancer, while downregulated in esophageal and other cancers. ITPR3 was versatile up-regulated in most cancers besides pancreatic cancer. By assembling the Cancer Cell Line Encyclopedia (CCLE), we found that all three members of ITPRs were up-regulated in pancreatic cancer cell lines. What is strikingly noticeable is that ITPR3 was the highest expressed in pancreatic cancer among all cell lines, implying its robust oncogenic function (Figure 1B).

Then, we investigated the expression of ITPRs in different microarray datasets (Table 1). In Segara’s dataset, ITPR1 was up-regulated with a fold change of 1.474 (*p*=0.03), and ITPR2 was up-regulated with a fold change of 1.235(*p*=0.013) [24]. Noteworthily, ITPR3 was significantly upregulated in pancreatic cancer patients in six datasets with low *p*-value much lower than 0.05, such as 1.83e^-7^, 6.03e^-8^ [24–29].

**Table 1:** The significant changes of ITPRs expression in the transcriptional level between cancer and normal tissues (Oncomine database).

**Gene ID**	**Types of pancreatic cancer versus normal**	**Fold change**	***P* Value**	**t test**	**References**
ITPR1	Pancreatic Carcinoma versus Normal	1.474	0.030	2.033	Segara [24]
	Pancreatic Ductal Adenocarcinoma vs. Normal	1.749	1.36e^-5^	4.616	Badea [25]
ITPR2	Pancreatic Carcinoma versus Normal	1.235	0.013	2.479	Segara [24]
ITPR3	Pancreatic Carcinoma versus Normal	2.462	3.99e^-4^	4.197	Segara [24]
	Pancreatic Ductal Adenocarcinoma versus Normal	1.948	1.12e^-9^	6.864	Badea [25]
	Pancreatic Adenocarcinoma versus Normal	16.110	0.045	2.199	Logsdon [26]
	Pancreatic Adenocarcinoma versus Normal	4.479	1.83e^-7^	8.568	Iacobuzio-Donahue [27]
	Pancreatic Ductal Adenocarcinoma versus Normal	2.314	0.034	1.925	Grutzmann [28]
	Pancreatic Carcinoma versus Normal	3.467	6.03e^-8^	6.784	Pei [29]

**Figure 2:**
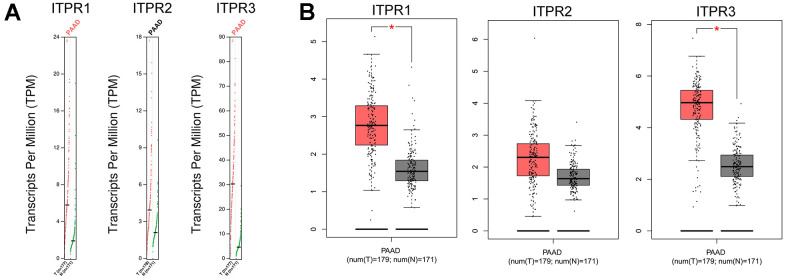
**The Expression of ITPRs family in pancreatic cancer (GEPIA).** (**A**) The Gene Expression Profiles of ITPR1, ITPR2, and ITPR3. (**B**) The Box plots of ITPR1, ITPR2, and ITPR3 genes Expressions.

Next, we further confirmed these results by using the GEPIA dataset. The result showed that ITPR1 and ITPR3 were up-regulated in pancreatic cancer specimens than non-tumor samples, confirming the previous results (Figure 2). Collectively, a desire is needed to value the survival value for ITPRs in pancreatic cancer.

### Up-regulated ITPR families predict poor clinical outcomes in pancreatic cancer patients

**Figure 3:**
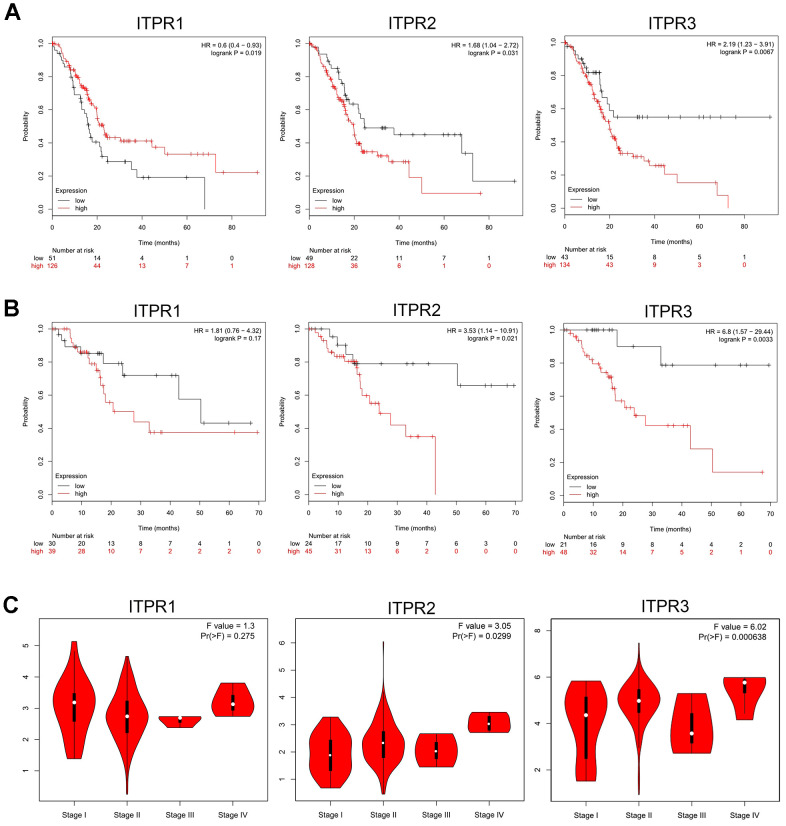
**Prognostic value of ITPRs in pancreatic cancer.** (**A**) The prognostic value of ITPRs in pancreatic cancer OS; (Kaplan-Meier Plotter database). (**B**) The prognostic value of ITPRs in pancreatic cancer RFS; (Kaplan-Meier Plotter database). (**C**) The prognostic value of ITPRs in pancreatic cancer staging. (GEPIA database).

To evaluate the function of ITPRs on the cancer patient’s clinicopathological and survival, we investigated survival analysis of ITPR1, ITPR2, and ITPR3 for pancreatic cancer by using Kaplan-Meier Plotter and GEPIA databases. As Kaplan-Meier Plotter analysis (Figure 3A) demonstrated, decreased expression of ITPR2 and ITPR3 indicated better overall survival for patients with pancreatic cancer. However, the elevated ITPR1 benefits overall survival, contradicting its expression results. We proposed that many censored patients may mislead the positive effect of ITPR1 in patients’ survival. This issue should be verified further. Meanwhile, lower expression of ITPR2 and ITPR3 indicated a relatively higher recurrence-free survival (Figure 3B), which confirmed the previous results. Furthermore, we verified these results by using the GEPIA database. Up-regulated ITPR2 and ITPR3 were associated with higher pathological stages of pancreatic cancer. Hence, it is advisable to indicate that ITPR2/3 predicts patients’ survival.

To further determine the association between clinical outcomes and ITPRs expression in TCGA datasets, we performed univariate and multivariate Cox logistic regression analyses (Table 2). In the univariate model, tumor grade (hazard ratio [HR] = 1.114; 95% confidence interval [CI] = 1.0200–1.8594; p =0.0366), ITPR1 expression (hazard ratio [HR] = 0.8443; 95% confidence interval [CI] = 0.7239–0.9847; p =0.0310), and ITPR3 expression (HR = 1.0247; 95% CI = 1.0077–1.0420, p =0.0042) were significantly correlated with OS. However, in multivariate Cox regression analysis showed that only the ITPR3 expression (HR = 1.0186; 95% CI = 1.0003-1.0372; p = 0.0452) were independent prognostic factors in the PAAD cohort from TCGA.

**Table 2:** Univariate and multivariate Cox logistic regression analysis of OS in The Cancer Genome Atlas (TCGA) cohorts.

**Covariates**	**Univariate analysis**			**Multivariate analysis**		
	**HR**	**95%CI**	**P value**	**HR**	**95%CI**	**P value**
Age	1.3543	0.8405-2.1821	0.2126	-	-	-
Gender	1.114	0.7309-1.6993	0.6143	-	-	-
Tumor Grade	1.3772	1.0200-1.8594	0.0366	1.2353	0.8949-1.7051	0.1988
Clinical Stage	1.4221	0.9787-2.0663	0.0646	-	-	-
ITPR1	0.8443	0.7239-0.9847	0.0310	0.8889	0.7570-1.0437	0.1506
ITPR2	0.9719	0.9080-1.0404	0.4131	-	-	-
ITPR3	1.0247	1.0077-1.0420	0.0042	1.0186	1.0003-1.0372	0.0452

### Alterations, immune infiltration, and methylations analysis for ITPRs

**Figure 4:**
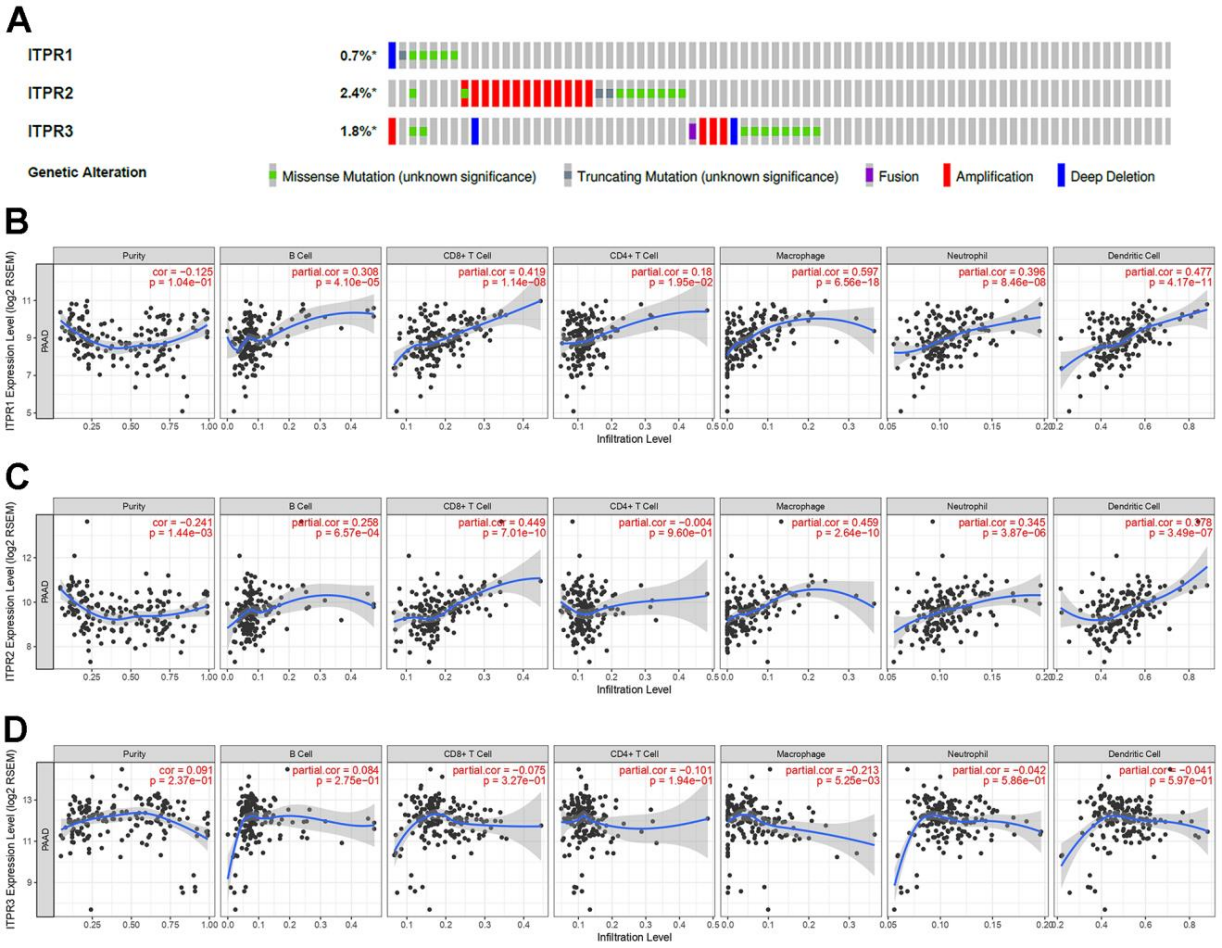
**The alterations and immune infiltration of ITPRs in pancreatic cancer.** (**A**) The alterations of ITPRs in pancreatic cancer (cBioPortal database). (**B**) The immune infiltration of ITPR1. (Timer database). (**C**) The immune infiltration of ITPR2. (Timer database). (**D**) The immune infiltration of ITPR3. (Timer database).

**Figure 5:**
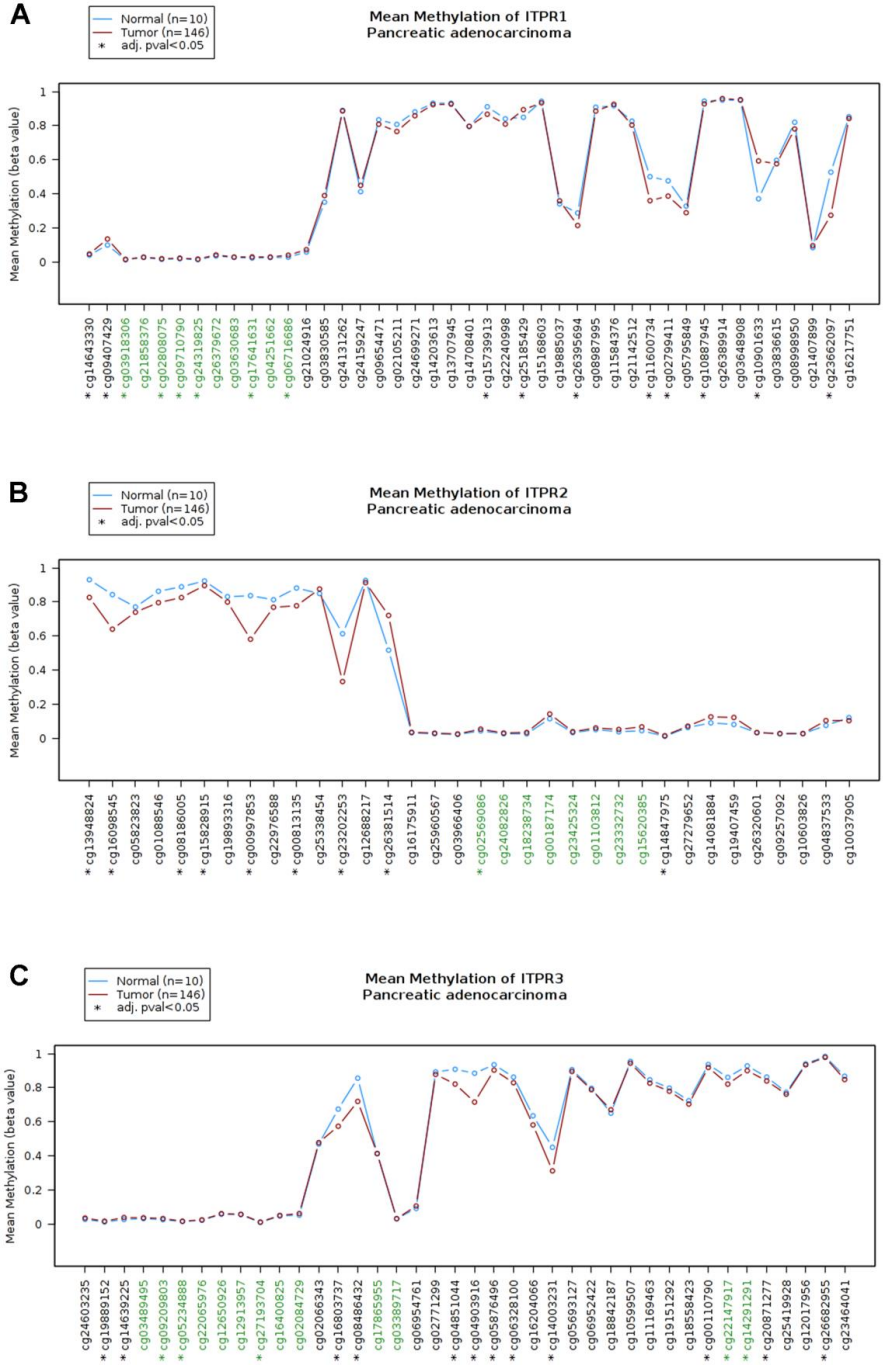
**The methylation status of ITPRs (Wanderer database).** (**A**) The methylation status of ITPR1. (**B**) The methylation status of ITPR2. (**C**) The methylation status of ITPR3.

The results above shown ITPRs family may play oncogene role in pancreatic cancer, but how did them work? Therefore, we analyzed the ITPRs alterations, As presented in Figure 4A, each of ITPRs has different degrees of mutations besides fusion, amplification and deletion, which may response for their cancer genetic. Immune infiltration and methylations are all distinctive phenomenon in human cancers. Next, we explored the whether ITPRs influence these aspects of pancreatic cancer. As presented in Figures 4B, ITPR1 expression level was positive correlated with infiltrating levels of B cells (r = 0.308, P = 4.10e-5), CD8+ T cells (r = 0.419, P = 1.14e-8), CD4+ T cells (r= 0.18, P = 1.95e-2), macrophages (r = 0.597, P = 2.14e-30), neutrophils (r = 0.396, P = 8.46e-8) and dendritic cells (r =0.477, P = 4.17e-11), while negatively correlated with tumor purity(r = -0.125, P = 1.04e-1). ITPR2 expression level was positive correlated with infiltrating levels of B cells (r = 0.258, P = 6057e-4), CD8+ T cells (r = 0.449, P = 7.01e-10), macrophages (r = 0.459, P = 2.64e-10), neutrophils (r = 0.345, P = 3.87e-6) and dendritic cells (r =0.378, P = 3.49e-7), while negatively correlated with tumor purity(r = -0.241, P = 1.44e-3). ITPR3 expression level was negative correlated with infiltrating levels of CD4+ T cells (r= -0.101, P = 1.94e-1), macrophages (r = -0.213, P = 5.25e-3), while no significant correlations with tumor purity and infiltrating levels of B cells, CD8+ T cells, neutrophils and dendritic cells. So ITPRs-indicted immune disorders may play a great part in cancer progression. From Figure 5, many significant methylation sites such as cg1464330, cg09407439, cg03918306 and cg02808075 in ITPR1; the cg13948824, cg16098545 cg08186005 and cg15828915 in ITPR2; cg19889152, cg14639225, cg09209803 and cg05234888 in ITPR3 were found, indicated that methylations may contribute to the ITPRs overexpression in pancreatic patients.

### GGI, PPI analysis, and functional enrichment analysis for ITPRs

**Figure 6:**
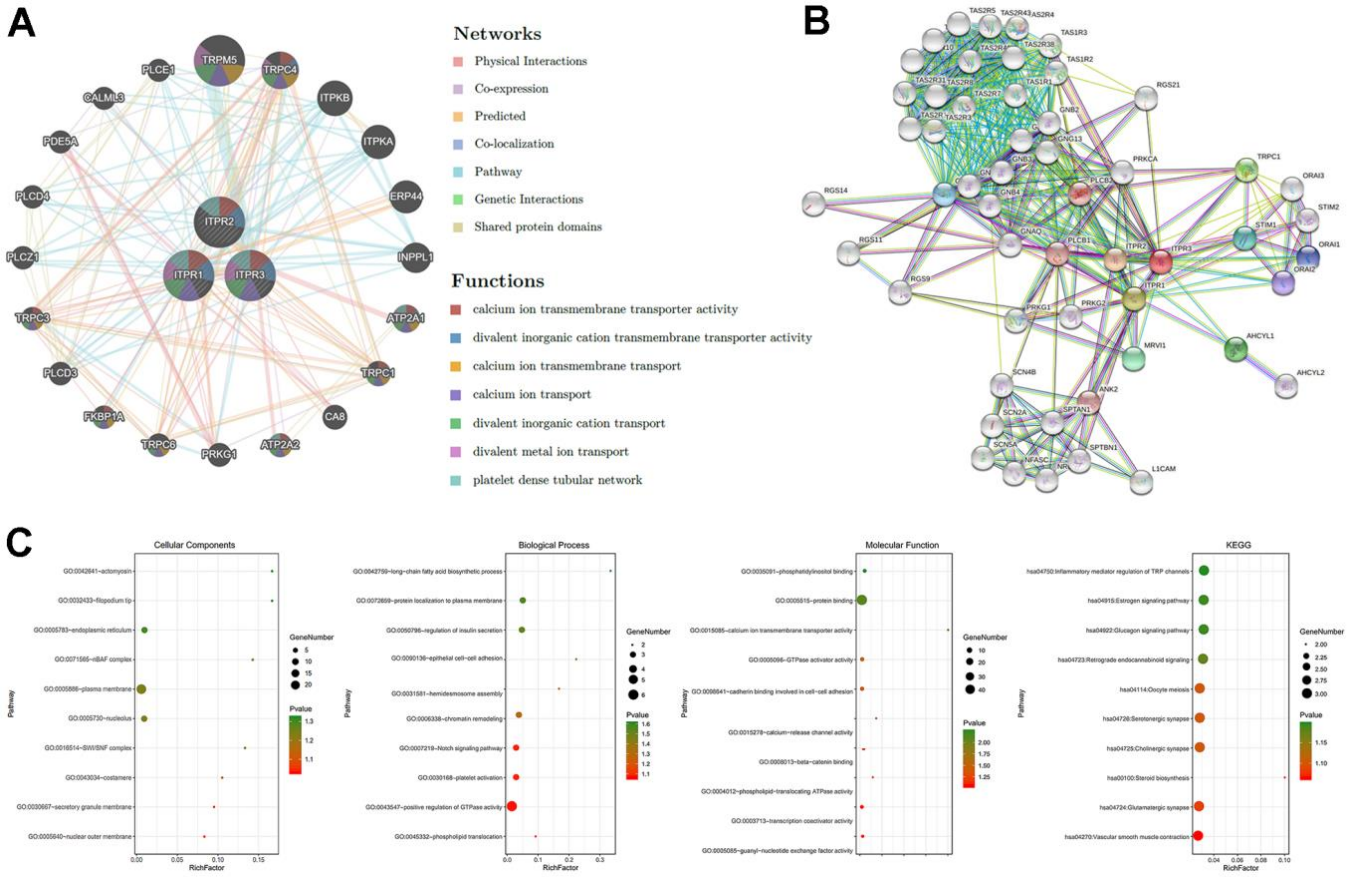
**The GGI, PPI, and predicted functions analysis of ITPRs.** (**A**) The GGI network of ITPRs (GeneMANIA). (**B**) The PPI network of ITPRs (String). (**C**) Predicted functions and pathways of ITPRs. (From left to right ITPR1, ITPR2, ITPR3) (David database).

Identifying more details about ITPRs could boost their potential function understanding. Next, the GGI network of three ITPRs was constructed using the GeneMANIA database in Figure 6A. These 20 represent genes positively connected with ITPRs by different manners such as shared protein domains, physical interactions, colocalization, and co-expression. The tops were TRPM5, TRPC4, ITPKB, ITPKA, ERP44. Next, we introduced the STRING database to construct a PPI network for ITPRs (Figure 6B). As a result, we found that ITPR1, ITPR2, and ITPR3 proteins may interact with tumor-promoting genes, such as PLCB1, PLCB2, PRKCA, and RGS21, which may partially explain its oncogene roles. The altered or abnormal expression of ITPRs may correlate with many different targets in cancer tissue, promote cancer initiation and progression, then influence the patients’ survival. Finally, we explored the functional enrichments of ITPRs and their neighboring genes by Gene Ontology (GO) and Kyoto Encyclopedia of Genes and Genomes pathways (KEGG) in the DAVID database (Figure 6C). The GO enrichment analysis consisted of three members: cellular component, biological process, and molecular function. The functions of the ITPRs family, such as cadherin binding involves in cell-cell adhesion, epithelial cell-cell adhesion, may influence tumor invasion; positive regulation of GTPase activity, endoplasmic reticulum, Notch signaling pathway, and phospholipid-translocating ATPase activity may promote cancer initiation and progression.

### Experimental verification of ITPRs in pancreatic cancer samples and cell lines

**Figure 7:**
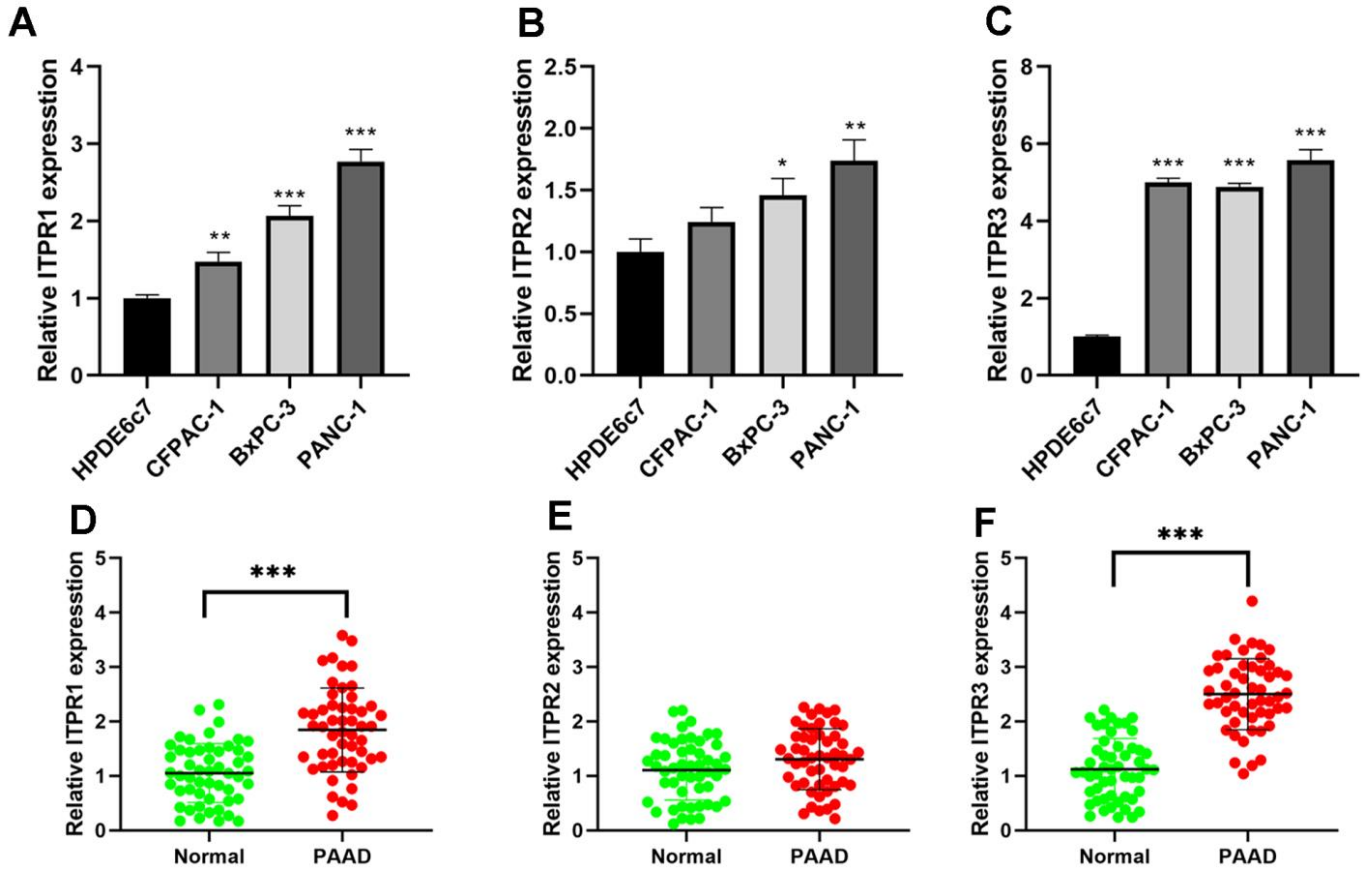
**The expression levels of ITPRs in pancreatic cancer samples and cell lines.** (**A**) The expression levels of ITPR1 in pancreatic cancer cell lines. (**B**) The expression levels of ITPR2 in pancreatic cancer cell lines. (**C**) The expression levels of ITPR3 in pancreatic cancer cell lines. (**D**) The expression levels of ITPR1 in pancreatic cancer samples. (**E**) The expression levels of ITPR2 in pancreatic cancer samples. (**F**) The expression levels of ITPR3 in pancreatic cancer samples.

ITPRs expression patterns were further validated in pancreatic cancer cell lines (BxPC-3, CFPAC-1, and PANC-1) and normal human pancreatic ductal cells (HPDE6c7) using qRT-PCR. In Figure 7A–7C, the ITPR1 was most up-regulated in BxPC-3 and PANC-1 than normal, while ITPR3 was in CFPAC-1 and PANC-1 cell lines. Then, we investigated ITPR1, ITPR2, and ITPR3 transcription levels in paired human PAAD tissue specimens and their corresponding nontumorous tissue samples. The results showed that ITPR1 and ITPR3 mRNA expression was markedly elevated in PAAD tissues relative to their normal counterparts (Figure 7D–7F). The clinical feature of these patients and their ITPR3 value are summarized in Table 3. High ITPR3 expression was correlated with higher TNM stage and lymph node invasion.

**Table 3:** Relationship between ITPR3 expression and clinicopathologic characteristics of PAAD patients.

**Clinical characteristics**	**Total**	**ITPR3 expression**		**P-value**
		**Low**	**High**	
Age				0.299
≤ 65 years	28	10	18	
> 65 years	24	12	12	
Gender				0.786
Male	26	12	14	
Female	26	10	16	
Serum CA19-9 level				0.055
>37 U/ml	38	11	27	
≤37 U/ml	14	9	5	
Histologic differentiation				0.101
Well	13	8	5	
Moderate	23	7	16	
Poor	16	10	6	
TNM stage				0.035
I-II	23	13	10	
III-IV	29	8	21	
Lymph node invasion				0.038
Present	29	6	23	
Absent	23	11	12	

### ITPR3 is required for PAAD cell proliferation, migration, and invasion *in vitro*

**Figure 8:**
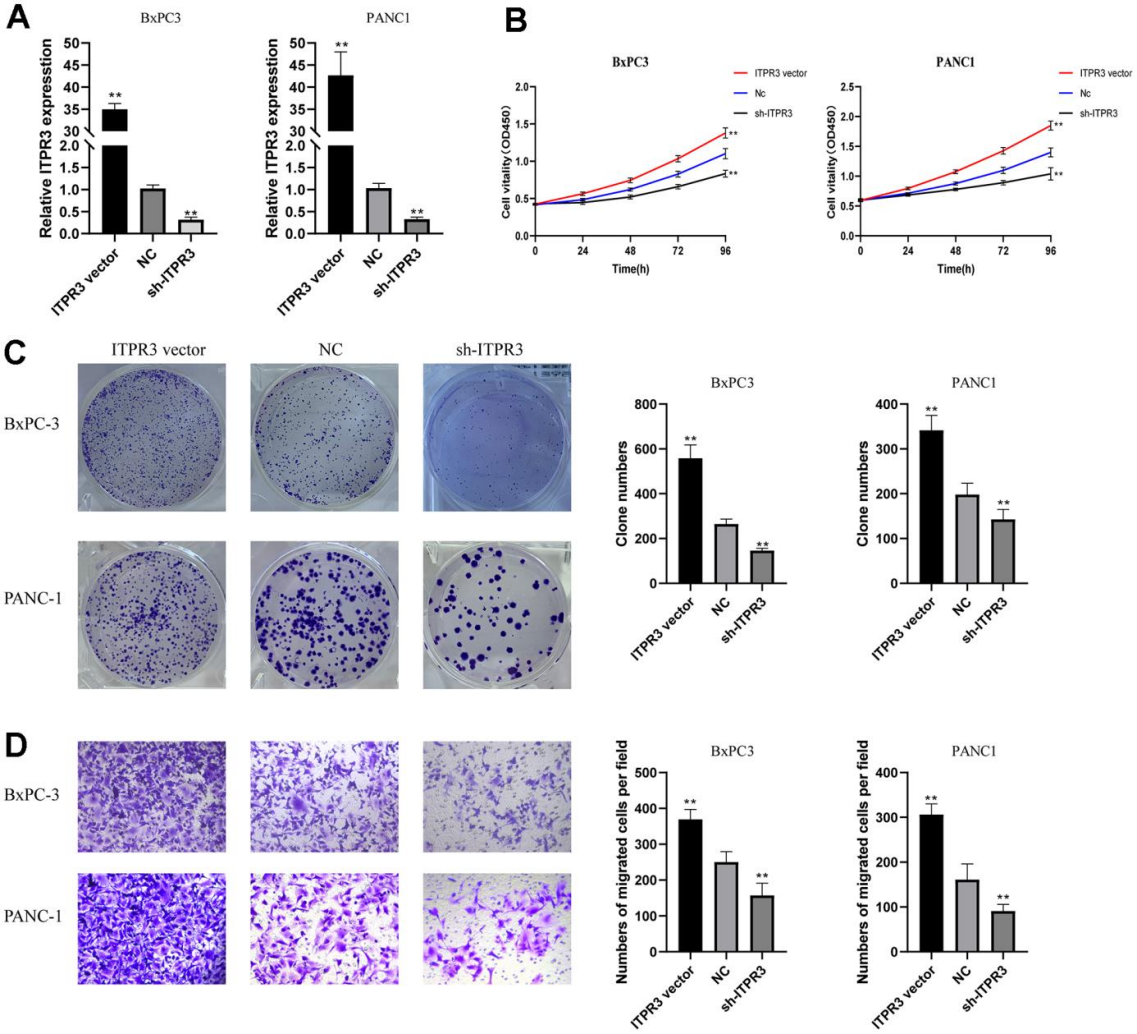
**ITPR3 is required for PAAD cell proliferation, migration, and invasion *in vitro*.** (**A**) RT-qPCR analysis was employed to examine the efficiency of ITPR3 knockdown or overexpression in Bxpc3 and PANC1 cells. (**B**) Proliferation curves were determined in ITPR3 knockdown or overexpressed Bxpc3 and PANC1 cells by cell counting kit-8 (CCK-8) assays. (**C**) Clonogenic assays measured colony-forming abilities in ITPR3 stable knockdown or overexpressed Bxpc3 and PANC1 cells. (**D**) Transwell assays were used to detect the migration and invasive capacities in ITPR3 stable knockdown or overexpressed Bxpc3 and PANC1 cells.

Given that ITPR3 is up-regulated in PAAD tissues and cell lines, it is necessary to determine whether the variable expression levels of ITPR3 could affect biologic activity in PAAD cells. We choose two high ITPR3 expressed cell lines BxPC-3, and PANC-1for further study. We stably downregulated ITPR3 expression using a lentivector carrying short hairpin RNA (shRNA) and up-regulated using lentiviral vectors encoding ITPR3. We tested these lentiviral vectors’ knockdown and overexpression efficiencies targeting ITPRs (Figure 8A). To explore the impact of ITPR3 repression on regulating cell proliferation, we performed cell counting kit-8 (CCK-8) and clone-forming assays. The proliferation curves determined by CCK-8 assays demonstrated that cell growth was remarkedly attenuated by ITPR3 knockdown in these cells. The clone-forming assays also showed that downregulation of ITPR3 in PAAD cells resulted in remarkably decreased cell growth and clonogenic ability. Conversely, overexpression of ITPR3 in these cells significantly increased cell proliferation and colony formation ability. (Figure 8B, 8C). We further explored the potential impact of ITPR3 on metastatic properties in PAAD cells by using transwell assays. In Figure 8D, transwell experiments revealed significantly decreased migration and invasive capabilities in the silencing ITPR3 groups while enhanced in the ITPR3 vector group. These results show that ITPR3 plays an essential part in PAAD cell proliferation, migration, and invasion *in vitro*.

### Effects of ITPR3 overexpression on endoplasmic reticulum stress (ERS)

**Figure 9:**
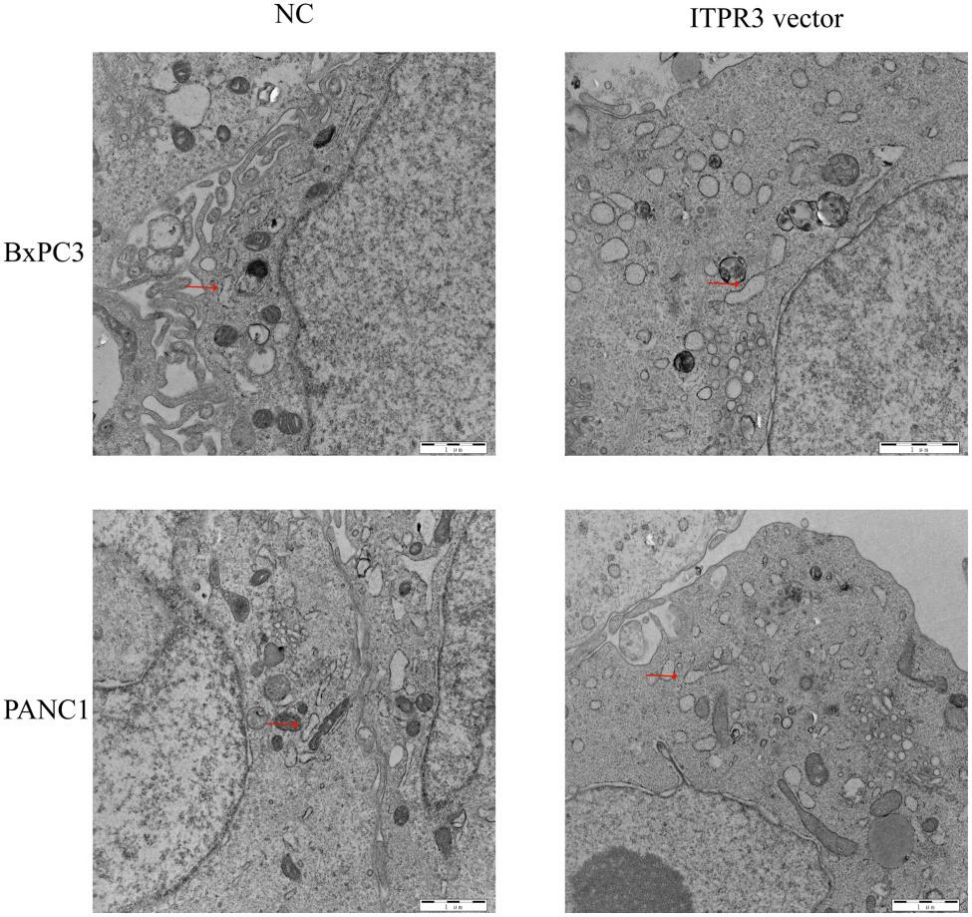
ITPR3 overexpression induces endoplasmic reticulum stress (ERS) in pancreatic cells.

The cells’ endoplasmic reticulum (ER) functioned as multiply cellular functions, such as protein biosynthesis, modifications or trafficking, and regulating Ca2+ homeostasis. As important intracellular Ca2+-release channels, the dysregulation of ITPR3 may be accompanied by the disorder of cell homeostasis and cause endoplasmic reticulum stress. As the effect of ITPR3 on ERS in PAAD cells remains unknown, we next investigated whether ITPR3 overexpression could induce ERS. The transmission electron microscope showed that ERS was observed in the cytoplasm of PAAD cells (Figure 9). Consistent with our predictions, the overexpression of ITPR3 would activate more ERS than the NC group, which is also compatible with the results of GO analysis.

## DISCUSSION

Pancreatic cancer, a common malignant neoplasm, is featured by high morbidity and mortality worldwide [2]. Pancreatic carcinogenesis is a combination of oncogenes, tumor-suppressor genes, and related molecules disorders. Therefore, it is essential to reveal its pathogenesis and identify sensitive and specific molecular biomarkers. Growing evidence has demonstrated the critical roles of ITPRs in cancers [15, 19–21]. The present study is the first to explore the expression, prognostic and potential target of ITPRs in pancreatic cancer.

ITPR1 is not only associated with neurological syndromes but also takes part in tumor carcinogenesis. ITPR1 works as a tumor suppressor in osteosarcoma and Sézary Syndrome [15, 16]. Alfugham et al. observed that ITPR1-IgG–positive patients had wide cancer dissemination, implying its function in tumor migration [30]. Then, ITPR1 was considered prominent in regulating renal cancer cell resistance to NK-mediated lysis. [31–33] ITPR1 is a new direct target of hypoxia-inducible factors 2a (HIF2a). Silencing ITPR1 in renal cancer cells inhibited NK-induced Autophagy and vice versa *in vivo* [33], And ITPR1 is involved in regulating intracellular calcium signaling and the regulation of Autophagy [34]. ITPR1 was also a key candidate gene in papillary thyroid carcinoma [35]. In Hu’s study, three genes (ITPR1, CCL2, and CDKN2A) were selected to predict papillary thyroid carcinoma patients’ survival [36]. PAAD Patients often have a hypodense image in contrast-enhanced computed tomography examination, while some exhibit hyperdense images. The last one has a higher micro vessel density, better prognosis, and is more suitable for anti-angiogenic therapy than the former. Xu et al. found high ITPR1 level may contribute to higher microvessel density, which results in the hyperdense image [37]. ITPR1 was also elevated in ovarian endometriosis tissues than normal. Knockdown may inhibit OE cells proliferation and induced apoptosis. Using bioinformatics technology, the author found camptothecin may be the effective drug to target ITPR1 and then validated it *in vivo* [38]. ITPR1 was found downregulated in the esophageal adenocarcinoma (EAC) tissues compared with the normal [39]. Basing the TCGA database, Zhao et al. found that ITPR1 could be a potential biomarker for esophageal adenocarcinoma [40]. ITPR1 may also involve in head and neck squamous cell carcinoma cancer genetics. Three genes (including ITPR1) survival models could predict HNSCC patient survival [41]. ITPR1 was also significantly down-regulated in breast cancer tissues compared with non-cancerous tissues. And has a high AUC value in breast cancer diagnosis [42]. Recently, induced by lncRNA EGOT, up-regulation of ITPR1 expression was also found can sensitize cancer cells to paclitaxel toxicity [43]. Great functions have now ITPR1 be proved; more investigations are still required to evaluate its diverse functions in multiply cancers. ITPR2 was found to participate in acute myeloid leukemia and clear cell renal cell carcinoma progression [17, 18]. As early as 1996, up-regulated ITPR3 has been proved to play an essential role in T lymphocytes apoptosis [44]. ITPR3 overexpression is correlated with bad clinic outcomes in cervical squamous cell carcinoma cancer [19], glioblastoma [20], cholangiocarcinoma [21]. ITPR3 was absent in normal colonic tissue while overexpressed in colorectal cancer [45]. And high ITPR3 expression was associated with poor 5-year overall survival. The following study showed that increased levels of ITPR3 give cancer cells a survival advantage over normal by consisting of apoptotic cell death. ITPR3 was also overexpressed in both CCA tissues and cell lines compared to histologically normal. ITPR3 in CCA could both inhibit cell death and promote proliferation invasiveness [21]. When deleted ITPR3, mitochondrial Ca2+ signaling in CCA cells was impaired, which induced cell necrotic death. Overexpression of ITPR3 also participates in the pathogenic mechanism of hepatocellular carcinoma (HCC) [46]. And become an independent predict indicator of HCC five-year survival. Recently, Wu et al. found that the independent SNP in ITPR3 (rs116454384C>T) has a better OS to predict the value in non-small cell lung cancer patients (hazards ratios of 0.85). But more mRNA expression levels of ITPR3 in blood and normal lung tissue were associated with better survival of NSCLC patients [47]. In bladder cancer tissues and cancer cells, ITPR3 was also highly expressed, resulting from the demethylation of the ITPR3 promoter region [48]. Overexpressed ITPR3 accelerates bladder cells cycle transformation and promotes invasion and metastasis by inducing epithelial-to-mesenchymal transition (EMT) progress. Furthermore, ITPRs maintained bladder cell stemness by CD44 then regulated the NF-κB signal pathway. These findings strongly support the vital roles of all ITPRs in carcinogenesis.

Earlier diagnosis of pancreatic cancer may lead to better patient outcomes. As detection methodologies improved, such as droplet digital PCR and Next Generation Sequencing technologies, liquid biopsy has increased. Liquid biopsy, such as ctDNA (circulating-free DNA), EVs (exosomes), and CTCs (circulating tumor cells) in bodily fluids such as blood, urine, and saliva, represented a rapid and noninvasive alternative to tissue biopsy represents to detect tumors release components, [49]. Plasma ctDNA was correlated with worse progression-free survival and OS [50, 51]. Moreover, the plasma ncRNAs miR-155-5p expression was directly associated with chemoresistance of gemcitabine and poor prognosis in PAAD [52]. The T cell receptor (TCR) is also an important biomarker for dictating antigen specificity of the T-cell mediated immunity [53]. Originated from multivesicular bodies, exosomes are nanosized single-membrane vesicles (30–150 nm), secreted by all cell types and exist in all body fluid types [54]. Exosomes reprogram other cells by cell-to-cell communication. And their lipid bilayer protects their cargo from degradation. Further research for studying ITPRs expression in ctDNA or exosome may take them into clinical.

Immunotherapy is a revolutionary treatment for many cancer types. Unfortunately, PAAD often exhibits no responses to immune checkpoint inhibitors treatment. PAAD has a low infiltration of tumor CD8+ T cells and a highly immunosuppressive microenvironment, making itself a cold tumor resistant to ICIs therapy [55]. And more tumor T cells are a favorable prognostic feature in PAAD. The crosstalk of how PAAD tumor cells evade the immune system is unclear. Our research found that ITPR1 and ITPR2 expression levels were correlated with CD8^+^ T cells infiltration. Some further experiments may be helpful to explain the underlying mechanisms. Epigenetic regulation is a dynamic event that may impact gene expression in PAAD [56]. Many cells proliferation-related genes were differentially methylated during PAAD progression [57]. In this paper, we pointed out many methylation sites of ITPRs in PAAD. A further experiment may reveal their molecular mechanisms in PAAD pathogenesis in detail. ER stress is a common phenomenon in cancer cells. But ER stress has shown to be a double-edged sword for tumour progression: it both can promote cancer cells’ survival or growth [58] and lead cells to death [59]. We found overexpressed ITPR3 could induce ERS in PAAD cells in this article. Exogenous disturbances of ITPR3 balances in PAAD cells may impair cells’ survival or growth and cause programmed cell death. So, ITPR3 could be a potential target for PAAD treatment.

This study found that ITPR1 and ITPR3 were up-regulated in pancreatic cancer, while there was no significant change in ITPR2. Secondly, we used Kaplan-Meier Plotter and GEPIA databases to explore the prognostic value of ITPRs in pancreatic cancer: decreased ITPR2 and ITPR3 indicated better survival. At the same time, ITPR2 and ITPR3 expression was associated with the pancreatic pathological stage. Moreover, ITPR3 expression could be an independent risk factor for PAAD patient survival. We predicted the alteration, co-expressed genes, and potential protein target of ITPRs. Immune infiltration, methylation, and functional enrichment of ITPRs were also analyzed. Then, we evaluated these findings in PAAD patients suggested that ITPR3 may be a robust factor in pancreatic cancer pathogenesis may sever as new drug targets and promising prognostic biomarkers for high pancreatic pathological cancer stage and poor disease outcome. Furthermore, ITPR3 silencing caused tumour suppressive effects via reducing cell proliferation, migration, and invasion, while overexpressed one increased these cell functions. The electron microscope assay shows that overexpressed ITPR3 may induce more endoplasmic reticulum stress in PAAD cell lines. Following more *in vivo* validation of the findings should be further studied. Along with more laboratory investigations and clinical trials, ITPRs might be translated into clinical use. In summary, our study suggested that ITPR3 is a potential target and new biomarker for the prognosis of pancreatic cancer.

## MATERIALS AND METHODS

### Patients and specimens

The Pancreatic Cancer specimens and corresponding non-cancerous tissues (n=52) were harvested from patients at the Second Affiliated Hospital of Harbin Medical University. These fresh specimens were preserved in liquid nitrogen. None of the patients received radiotherapy, chemotherapy before surgery. This study was authorized by the Ethics Committee of the Second Affiliated Hospital of Harbin Medical University.

### Cell culture and cell transfection

The normal pancreatic cell lines HPDE6-C7 and cancer cell lines BxPC-3, CFPAC-1, PANC-1 were bought from the Shanghai Institute of Biological Science and Cell Resources Center, Chinese Academy of Sciences (Shanghai, China). The HPDE6-C7, CFPAC-1, and PANC-1 cells were cultured in DMEM medium containing 10% fetal bovine serum (FBS; HyClone Laboratories, Logan, UT), while BxPC-3 was cultured in RPMI-1640. And each medium was supplemented with 100 U/mL penicillin and 100 mg/mL streptomycin. All the cells were placed in an incubator in line with typical culture conditions (37° C, 5% CO2). Small -hairpin RNA directed against ITPR3 were designed and synthesized by Gene Chem (Shanghai, China). The sequences are (5′- GAAGCAAGUUUGAGGAGAATT-3′ and 3′- UUCUCCUCAAACUUGCUUCTT-5′). An empty Sh-NC vector was used as a control. The sequences are (5′-UUCUCCGAACGUGUCACGUTT-3′ and 3′- ACGUGACACGUUCGGAGAATT-5′). Lentiviral vectors encoding ITPR3 were generated by (Gene Chem, Shanghai, China). The empty vector was used as a negative control. The procedure of lentiviral infection was conducted by the instructions of the manufacturer. The selection of qualified cells was performed by using puromycin for 3–4 weeks.

### RNA isolation and qRT-PCR

Total RNA from PAAD tissue specimens and cultured cells was isolated by TRIzol (Sigma, MO, USA), and then 1μg of RNA was applied to synthesize the complementary DNA (cDNA) with a First Strand cDNA Synthesis Kit (Roche, Germany). Specific gene expression was detected by using the FastStart Universal SYBR Green Master Kit (Roche, Germany). GAPDH was used for internal control of the expression of mRNA. Sequences of all of the genes primers are listed in Supplementary Table 1. The mRNA relative expression data were normalized and calculated using equation 2 -ΔΔCT.

### CCK-8 and colony-forming experiments

CCK-8 (Cell counting kit-8) (Dojindo, Japan) was employed to determine cells viability. A density of 3× 10^4^ cells per well was seeded in 96-well plates. 10 μl of reagent was added to each well and maintained for 2 h at 37° C. At 0, 24, 48, 72, and 96 h. Then cells were measured by the reader (Tecan, Switzerland) at a wavelength of 450 nm. One thousand cells per well were plated in six-well plates for colony formation assays. At 15 days after plating, the cells were fixed and stained.

### Migration and invasion assays

Transwell chambers (Corning, NY, USA) were applied to further access cellular motility. 4× 10^4^ cells were resuspended in 200μL of FBS-free DMEM or RPMI 1640 in the higher chambers while lower champers was placed with 600μL 10% FBS DMEM or RPMI 1640. After incubating for 24h at 37° C, cells on the upper surface of the chambers were eliminated. Then, the membranes were fixed and stained. The numbers of invasive or migrated cells were counted using a microscope.

### Transmission electron microscopy

ERS was examined by transmission electron microscopy**.** We harvested transfected BxPC-3 and PANC-1 cells then fixed them in glutaraldehyde. After being dehydrated by acetone, these embedded cells were cut into 60-nm ultrathin sections in Ultracut (Leica, Germany). After staining with uranyl acetate, the ultrathin sections were examined with a transmission electron microscope.

### Oncomine analysis and CCLE analysis

The genes expression in cancers was made by the ONCOMINE platform, a publicly accessible, well-maintained bioinformatics database (https://www.oncomine.com/), using a students’ t-test to generate a p-value. The genes expression rank in cancers cell lines was constructed by Cancer Cell Line Encyclopedia (CCLE) (https://www.broadinstitute.org/ccle). CCLE is an interactive database created by the Broad Institute and the Novartis Institutes.

### The Kaplan–Meier Plotter analysis

Cancer patients’ survival analysis was predicted by The Kaplan-Meier Plotter (http://kmplot.com/analysis/) and confirmed by GEPIA (Gene Expression Profiling Interactive Analysis) (http://gepia.cancer-pku.cn/). These tools are free open-access web which could date from the GEO (Gene Expression Omnibus), Cancer Genome Atlas (TCGA), and Genotype-tissue Expression dataset (GTEx). The Kaplan-Meier Plotter’s hazard ratio (HR) is 95% confidence intervals, and P-value was generated by computer [60]. GEPIA provides robust differential expression, patient survival date, and similarity gene detection [61].

### cBioPortal analysis

The gene alteration was analyzed by the cBioPortal database. (http://www.cbioportal.org/) which was created by Memorial Sloan Kettering Cancer Center, is an available online database for cancer research by analyzing gene alternation predicting patient survival and potential target [62, 63].

### Timer analysis

The immune infiltrations information was searched in the Timer database (https://cistrome.shinyapps.io/timer/). This website integrated diverse cancer immune infiltrations information is a reliable resource for immune infiltration analysis. The ITPRs’ immune features such as B cells, T cells, neutrophils, macrophages, and dendritic cells were created by this database [64].

### Wanderer analysis

The Wanderer database was employed to calculate the methylation status of the ITPRs gene. Wanderer (http://maplab.imppc.org/wanderer) is a website that contains gene methylation and gene expression data for multiply human cancers. The data is from TCGA (The Cancer Genome Atlas) project [65].

### GeneMANIA analysis

GeneMANIA (http://www.genemania.org/) is an open-access online analysis tool for exploring gene relationships networks. By querying a list of genes, GeneMANIA generates the relevant genes with similar functions to the target gene and constructs an interactive network [66]. In this study, we used GeneMANIA to build an interaction gene-gene network for ITPRs.

### STRINGS analysis

STRING (https://string-db.org/), a free online bioinformatics database, was used to predict and visualize data the interaction of protein and protein [67]. We used this database to predict protein-protein interactions.

### DAVID analysis

Visualization and Integrated Discovery (https://david.ncifcrf.gov/summary.jsp) is an online bioinformatics database for Gene Ontology (GO) and Kyoto Encyclopedia of Genes and Genomes (KEGG) enrichment analysis. The GO contains three aspects: biological process, cellular component, and molecular function.

### Statistical analysis

The TCGA data was downloaded by GDC (Genomic Data Commons) online tools. Multivariate Cox regression was analyzed using R software version 3.6.1’s RMS package to identify independent prognostic factors for pancreatic cancer. A p-value < 0.05 was considered to be statistically significant. GraphPad Prism 5.01 (GraphPad Software, CA, USA) was used to compare gene expression differences between cancer and noncancer samples.

### Data availability statement

The datasets presented in this study can be found in online repositories.

## Supplementary Material

Supplementary Table 1
