# Supplementary material for: Transcriptional ITPR3 as potential targets and biomarkers for human pancreatic cancer
Source: Aging (Albany NY). 2022 May 17;14(10):4425–44. doi: 10.18632/aging.204080 (PMC9186782; doi:10.18632/aging.204080)
Supplement: Supplementary Table 1 [file aging-14-204080-s001.pdf]

SUPPLEMENTARY TABLE

Supplementary Table 1. Primer sequences oligonucleotides information.

Gene/miRNAs	Sequence
ITPR1	Forward primer: 5′- GCGGAGGGATCGACAAATGG-3′<br>Reverse primer: 5′-TGGGACATAGCTTAAAGAGGCA-3′
ITPR2	Forward primer: 5′-CACCTTGGGGTTAGTGGATGA-3′<br>Reverse primer: 5′-CTCGGTGTGGTTCCCTTGT -3′
ITPR3	Forward primer: 5′-CCAAGCAGACTAAGCAGGACA-3′<br>Reverse primer: 5′-ACACTGCCATACTTCACGACA-3′
GAPDH	Forward primer: 5′- ATTTGCCTGCATTACCGGTC-3′<br>Reverse primer: 5′-ATCAACGTTTTCTTTTCGG-3′
